# Effect of Chronic Pioglitazone Treatment on Hepatic Gene Expression Profile in Obese C57BL/6J Mice

**DOI:** 10.3390/ijms160612213

**Published:** 2015-05-29

**Authors:** Chunming Jia, Yi Huan, Shuainan Liu, Shaocong Hou, Sujuan Sun, Caina Li, Quan Liu, Qian Jiang, Yue Wang, Zhufang Shen

**Affiliations:** State Key Laboratory of Bioactive Substances and Functions of Natural Medicines, Institute of Materia Medica, Chinese Academy of Medical Sciences and Peking Union Medical College, Beijing 100050, China; E-Mails: jiachunming@imm.ac.cn (C.J.); tomboyyi@imm.ac.cn (Y.H.); liusn@imm.ac.cn (S.L.); shaoconghou@imm.ac.cn (S.H.); sunsj@imm.ac.cn (S.S.); leecaina@imm.ac.cn (C.L.); popliu@imm.ac.cn (Q.L.); jiangqian@imm.ac.cn (Q.J.); wangyue@imm.ac.cn (Y.W.)

**Keywords:** pioglitazone, non-alcoholic fatty liver disease, Affymetrix Mouse GeneChip, inflammation response, lipid metabolism

## Abstract

Pioglitazone, a selective ligand of peroxisome proliferator-activated receptor gamma (PPARγ), is an insulin sensitizer drug that is being used in a number of insulin-resistant conditions, including non-alcoholic fatty liver disease (NAFLD). However, there is a discrepancy between preclinical and clinical data in the literature and the benefits of pioglitazone treatment as well as the precise mechanism of action remain unclear. In the present study, we determined the effect of chronic pioglitazone treatment on hepatic gene expression profile in diet-induced obesity (DIO) C57BL/6J mice in order to understand the mechanisms of NAFLD induced by PPARγ agonists. DIO mice were treated with pioglitazone (25 mg/kg/day) for 38 days, the gene expression profile in liver was evaluated using Affymetrix Mouse GeneChip 1.0 ST array. Pioglitazone treatment resulted in exacerbated hepatic steatosis and increased hepatic triglyceride and free fatty acids concentrations, though significantly increased the glucose infusion rate in hyperinsulinemic-euglycemic clamp test. The differentially expressed genes in liver of pioglitazone treated *vs.* untreated mice include 260 upregulated and 86 downregulated genes. Gene Ontology based enrichment analysis suggests that inflammation response is transcriptionally downregulated, while lipid metabolism is transcriptionally upregulated. This may underlie the observed aggravating liver steatosis and ameliorated systemic insulin resistance in DIO mice.

## 1. Introduction

Non-alcoholic fatty liver disease (NAFLD) is a cluster of liver disorders marked by hepatic lipid accumulation (steatosis) in the absence of other pathologies such as hepatitis infection or alcohol abuse [1]. It is perhaps the most common of all liver disorders as it affects 10% to 35% of the population in several countries [2]. Importantly, increasing clinical and epidemiological evidence suggests that NAFLD is associated not only with liver-related morbidity and mortality, but also with an increased risk of developing both cardiovascular disease and type 2 diabetes mellitus (T2DM) [3]. Insulin resistance is critical in the pathogenesis of NAFLD by inducing an imbalance between factors that exacerbate hepatic lipid accumulation, such as *de novo* lipid synthesis and lipid influx, and factors that ameliorate lipid build-up, such as lipid export or oxidation [4]. Thiazolidinedione (TZD) antidiabetic agents could reverse the abnormalities by improving insulin resistance, which have been used in clinical studies to prevent the progression of NAFLD [5,6,7].

TZDs act via peroxisome proliferator activated receptor gamma (PPARγ), a nuclear receptor, which is most highly expressed in fat tissues and regulates genes involved in fatty acid uptake and storage, inflammation, and glucose homeostasis [8]. In contrast to healthy liver, up-regulation of PPARγ expression in liver was a feature of obese animals, including KKAy mice [9], ob/ob mice [10,11], db/db mice [11], as well as lipoatrophic A-Zip/F1 mice [12], all of which developed severe hepatic steatosis. Accordingly, targeted deletion of PPARγ in hepatocytes and macrophages protected mice against diet-induced hepatic steatosis [13], suggesting a pro-steatotic role of PPARγ both in parenchymal and non-parenchymal cells. Moreover, ob/ob mice treated with rosiglitazone did not reverse histological NAFLD, but rather had increased oxidative stress and liver steatosis [14,15]. In contrast to these studies showing a deleterious effect of PPARγ on NAFLD, adenovirus-mediated overexpression of PPARγ in methionine-choline deficient (MCD) diet-induced models of NAFLD were shown to improve hepatic steatosis, inflammation and fibrosis [16]. In line, heterozygous PPARγ-deficient mice developed more severe MCD-induced NAFLD, whereas treatment with PPARγ agonists, including rosiglitazone and pioglitazone, prevented the development of NAFLD with fibrosis [16,17,18,19]. Similar findings were also observed in male low-density lipoprotein receptor (LDLR)^−/−^ mice fed a high-fat diet [18]. Although these findings and our own research in KKAy mice [20] all implied a strong relationship between NAFLD and PPARγ, the effects of PPARγ on hepatic steatosis have not been conclusive, as the administration of PPARγ agonists in different animal models induced seemingly opposing effects.

To our knowledge, no previous studies have analyzed the global expression profile of pioglitazone-treated DIO mice, which exhibit several metabolic and physiological similarities to human T2DM, such as obesity, hyperglycemia, insulin resistance, and dyslipidemia combined with apparent fatty liver [21]. The aim of this study is therefore to investigate if long-term treatment with pioglitazone affects global mRNA expression profile in DIO mice livers. Diverse bioinformatic analysis of microarray gene expression data was used to identify genes and biological processes contributing to the characteristic effect of PPARγ agonists on NAFLD.

## 2. Results

### 2.1. Pioglitazone Ameliorated Systemic Insulin Resistance 

**Figure 1:**
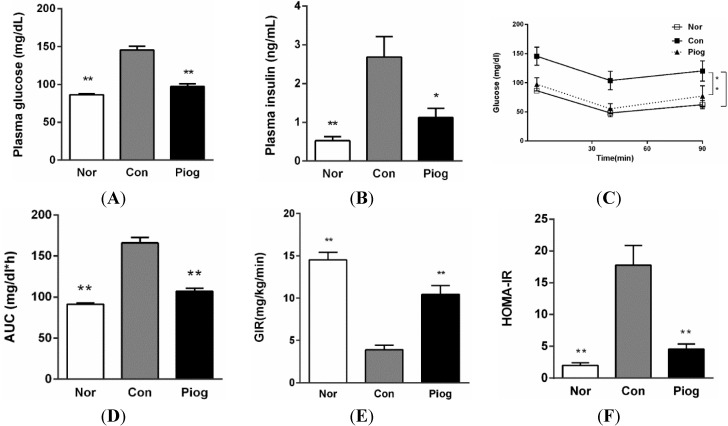
Pioglitazone treatment improved systemic insulin resistance in DIO mice. (**A**) Fasting blood glucose; (**B**) fasting blood insulin; (**C**) blood glucose levels in ITT; (**D**) areas under the curves of blood glucose in ITT (*n* = 12); (**E**) GIR (Glucose infusion rate) in hyperinsulinemic-euglycemic clamp test (*n* = 4); and (**F**) HOMA-IR was calculated by (FBG (mg/dL) × FINS (ng/mL))/22.5. Data are mean ± S.E.M., * *p* < 0.05, ** *p* < 0.01 *vs.* Con.

As shown in Figure 1, fasting blood glucose (FBG) and fasting blood insulin (FINS) levels in DIO mice were increased by 1.8 and 5.1 folds compared with those in the Normal mice (145.5 ± 4.2 *vs.* 86.1 ± 1.8 mg/dL, 2.7 ± 0.5 and 0.5 ± 0.2 ng/mL, *p* < 0.01), respectively. Moreover, results from hyperinsulinemic-euglycemic clamp and insulin tolerance test (ITT) demonstrated that DIO mice possessed extreme insulin insensitivity.

Compared to the control group, pioglitazone treatment significantly decreased the fasting plasma glucose and insulin levels by 32.7% and 58.0%, respectively (Figure 1A,B). It also improved the glucose curve and area under curve (AUC) perceived in ITT (Figure 1C,D) and the homeostasis model of assessment for insulin resistance index (HOMA-IR) (Figure 1F). The glucose infusion rate (GIR) from Hyperinsulinemic-euglycemic clamp, which is known as the golden standard for assessing insulin resistance, was significantly increased up to 169.0% in the group treated with pioglitazone. These findings indicated that pioglitazone can efficiently enhance systemic insulin sensitivity in DIO mice.

### 2.2. Pioglitazone Exacerbated Hepatic Steatosis

**Figure 2:**
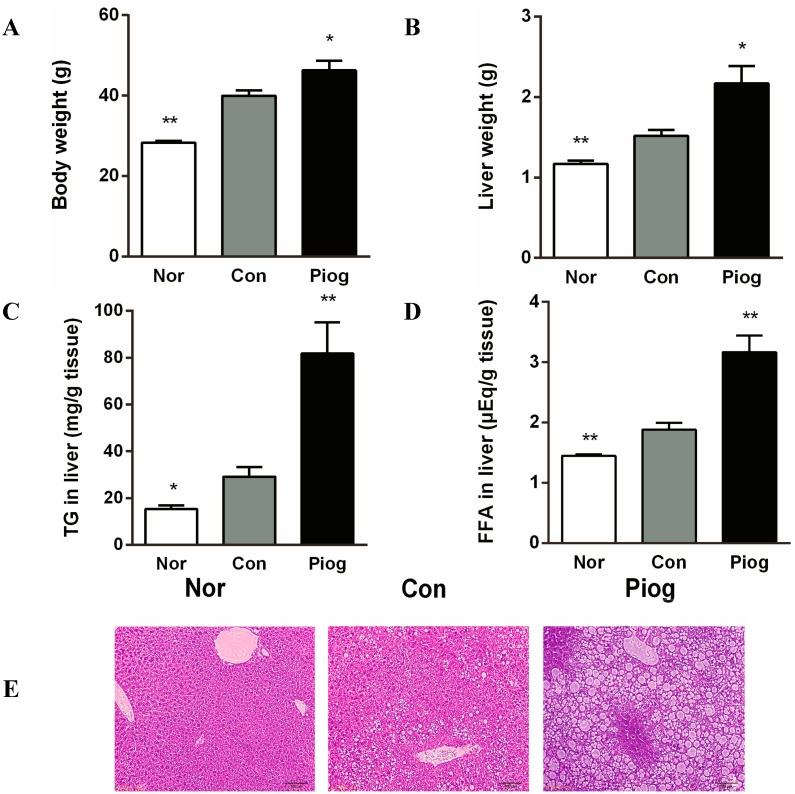
Effect of pioglitazone treatment on hepatic steatosis in DIO mice: (**A**) body weight; (**B**) liver weight; (**C**) liver triglyceride; (**D**) liver-free fatty acids; and (**E**) liver histology, hematoxylin and eosin staining, bar = 100 µm. Data are mean ± S.E.M. (*n* = 8), * *p* < 0.05, ** *p* < 0.01 *vs.* Con.

Body weight and liver weight in DIO mice were significantly increased compared to normal chow fed mice (Figure 2A,B). Biochemical analysis of hepatic lipid contents and histopathology also revealed that these obese mice developed manifest hepatic steatosis (Figure 2C–E). Following the 38-day treatment with pioglitazone, liver steatosis was markedly aggravated. The final body weight and liver weight in pioglitazone-treated DIO mice were increased by 15.8% (*p* < 0.05) and 42.8% (*p* < 0.05), respectively. Furthermore, the treatment increased liver triglyceride (TG) content from 29.2 ± 4.0 to 81.8 ± 13.3 mg/g liver (*p* < 0.01) and liver free fatty acids (FFA) content from 1.88 ± 0.1 to 3.16 ± 0.3 μEq/g tissue. This unexpected effect was confirmed by H&E staining of tissue samples (Figure 2E), showing increased lipid stores in pioglitazone treated mice. 

### 2.3. Identification and Classification of Differentially Expressed Genes by Microarray Analysis

**Figure 3:**
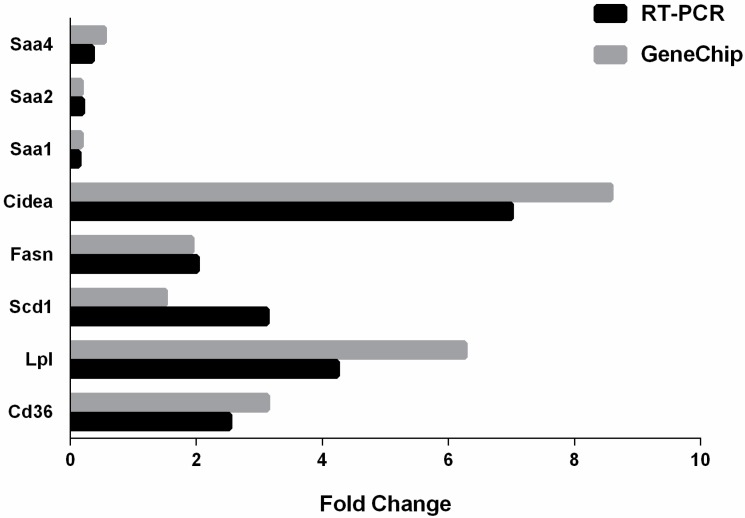
Validation of GeneChip data with RT-PCR. The expression of eight genes was analyzed using GeneChip and RT-PCR. The changes in expression of these genes were similar in the direction and magnitude between the two techniques.

Comparison analysis of the expression profiles was performed between Pioglitazone-treated mice and control mice from the Gene Chip data. Using a log2 fold change of ±1.5 and *p* < 0.05 as a cutoff, we identified a total of 346 genes that were differentially expressed in pioglitazone-treated livers compare to control livers. Among these 346 regulated genes, 260 transcripts were up- and 86 were downregulated. Microarray data were validated using qRT-PCR for eight selected DEGs, which demonstrated high correlation between microarray and qRT-PCR expression levels (Figure 3).

Functional enrichment analyses of the 346 differentially expressed genes were performed to identify over-represented biological functions using Gene Ontology terms and pathways. DAVID identified 242 and 82 differentially expressed genes in the up- and downregulated biological functions, respectively. Table 1 and Table 2 showed selected subsets of the over-represented biological functions and pathway; the upregulated genes were enriched in energy metabolism-related functions such as “lipid metabolic process”, “PPAR signaling pathway” and “oxidation reduction”, while downregulated genes were enriched in “inflammatory response”, “cytokine-cytokine receptor interaction” and “localization”.

As shown in Table 3, pioglitazone treatment upregulated RNA expression of fatty acid binding protein 4 (Fabp4), CD36 antigen (Cd36), fatty acid synthase (Fasn), which were all related to liver TG levels regulation. Meanwhile, pioglitazone treatment significantly decreased the expression of serum amyloid A (Saa) encoding a major acute-phase serum amyloid A protein.

**Table 1:** Over-represented biological functions analysis in differentially expressed genes in DIO mice treated with pioglitazone for 38 days.

	Biological Function	Gene Count	Benjamini	Fold Enrichment
**Upregulated in Progressors**	lipid metabolic process	33	6.50 × 10^−7^	3.6
	cellular ketone metabolic process	27	9.00 × 10^−7^	4.2
	carboxylic acid metabolic process	26	1.70 × 10^−6^	4.1
	oxoacid metabolic process	26	1.70 × 10^−6^	4.1
	organic acid metabolic process	26	1.30 × 10^−6^	4.1
	monocarboxylic acid metabolic process	19	4.00 × 10^−6^	5.4
	cellular lipid metabolic process	25	4.50 × 10^−6^	3.9
	fatty acid metabolic process	15	2.70 × 10^−5^	6.2
	oxidation reduction	25	1.30 × 10^–3^	2.8
	lipid biosynthetic process	15	3.60 × 10^−3^	4
**Downregulated in Progressors**	acute inflammatory response	7	7.60 × 10^−4^	20.1
	acute-phase response	5	2.50 × 10^−3^	38.8
	inflammatory response	8	9.70 × 10^−3^	8.3
	defense response	9	9.00 × 10^−2^	4.7
	response to wounding	8	8.10 × 10^−2^	5.4
	response to external stimulus	10	1.40 × 10^−1^	3.6
	fat cell differentiation	4	1.90 × 10^−1^	15.3
	localization	21	3.40 × 10^−1^	1.8
	transport	19	3.90 × 10^−1^	1.9
	brown fat cell differentiation	3	3.60 × 10^−1^	24.1

The over-represented top 10 biological functions regulated in differentially expressed genes are listed.

**Table 2:** KEGG pathway analysis of gene set over-representation in differentially expressed genes in DIO mice treated with pioglitazone for 38 days.

Term	Gene Count	Benjamini	Fold Enrichment
**Upregulated**			
PPAR signaling pathway	12	5.80 × 10^−6^	9.1
drug metabolism	11	1.80 × 10^−5^	8.8
fatty acid metabolism	8	2.80 × 10^−4^	10.6
metabolism of xenobiotics by cytochrome P450	9	3.10 × 10^−4^	8.2
Retinol metabolism	9	3.10 × 10^−4^	7.9
valine, leucine and isoleucine degradation	6	1.70 × 10^−2^	7.8
glutathione metabolism	6	2.50 × 10^−2^	6.9
arachidonic acid metabolism	7	3.30 × 10^−2^	5
pyruvate metabolism	5	5.50 × 10^−2^	7.3
biosynthesis of unsaturated fatty acids	4	1.00 × 10^−1^	8.9
**Downregulated**			
drug metabolism	3	8.60 × 10^−1^	9.6
cytokine-cytokine receptor interaction	4	8.70 × 10^−1^	3.9

DAVID identified 96 and 24 over-represented differentially expressed genes among the up- and downregulated KEGG pathway. The over-represented top 10 upregulated and 2 downregulated of KEGG pathway are listed.

**Table 3:** Differentially expressed genes related to lipid metabolic process, PPAR signaling pathway (upregulated) and inflammatory response (downregulated) in DIO mice treated with pioglitazone for 38 days.

Gene Symbol	Description	*p*-Value	Fold-Change
**PPAR Signaling Pathway**			
*Cd36*	CD36 antigen	1.54 × 10^−4^	3.13
*Acaa1b*	acetyl-Coenzyme A acyltransferase 1B	2.65 × 10^−5^	1.79
*Acsl5*	acyl-CoA synthetase long-chain family member 5	1.90 × 10^−4^	1.5
*Cpt1b*	carnitine palmitoyltransferase 1b, muscle	5.93 × 10^−3^	1.67
*Cyp4a14*	cytochrome P450, family 4, subfamily a, polypeptide 14	2.50 × 10^−6^	3.58
*Ehhadh*	enoyl-Coenzyme A, hydratase/3-hydroxyacyl Coenzyme A dehydrogenase	5.70 × 10^−6^	1.95
*Fabp4*	fatty acid binding protein 4, adipocyte	3.04 × 10^−4^	6.39
*Lpl*	lipoprotein lipase; similar to Lipoprotein lipase precursor (LPL)	3.64 × 10^−5^	6.26
*Pltp*	phospholipid transfer protein	6.42 × 10^−5^	2.37
*Me1*	malic enzyme 1, NADP(+)-dependent, cytosolic	9.10 × 10^−6^	2.25
*Cyp4a31*	cytochrome P450, family 4, subfamily a, polypeptide 31;	1.03 × 10^−5^	1.86
*Cyp4a10*	cytochrome P450, family 4, subfamily a, polypeptide 10;	3.92 × 10^−5^	1.75
*Cyp4a32*	cytochrome P450, family 4, subfamily a, polypeptide 32;	4.59 × 10^−5^	1.83
*Scd1*	stearoyl-Coenzyme A desaturase 1	1.11 × 10^−4^	1.51
**Lipid Metabolic Process**			
*Agpat9*	1-acylglycerol-3-phosphate *O*-acyltransferase 9	1.80 × 10^−3^	1.7
*Cd74*	CD74 antigen (invariant polypeptide of major histocompatibility complex, class II antigen-associated)	2.75 × 10^−3^	1.62
*Elovl5*	ELOVL family member 5, elongation of long chain fatty acids (yeast)	6.05 × 10^−5^	2.05
*Elovl7*	ELOVL family member 7, elongation of long chain fatty acids (yeast)	1.12 × 10^−2^	2.1
*9130409I23Rik*	RIKEN cDNA 9130409I23 gene	1.33 × 10^−4^	2.63
*Acot2*	acyl-CoA thioesterase 2	2.90 × 10^−3^	1.93
*Acer2*	alkaline ceramidase 2	1.63 × 10^−3^	1.77
*Crat*	carnitine acetyltransferase	1.88 × 10^−3^	1.68
*Cidea*	cell death-inducing DNA fragmentation factor, α subunit-like effector A	3.01 × 10^−3^	8.58
*Cyp17a1*	cytochrome P450, family 17, subfamily a, polypeptide 1	7.22 × 10^−3^	2.7
*Elovl3*	elongation of very long chain fatty acids (FEN1/Elo2, SUR4/Elo3, yeast)-like 3	3.52 × 10^−4^	1.83
*Ebpl*	emopamil binding protein-like	6.39 × 10^−5^	1.51
*Fasn*	fatty acid synthase	4.53 × 10^−4^	1.93
*Far2*	fatty acyl CoA reductase 2	3.78 × 10^−3^	1.73
*Osbpl3*	oxysterol binding protein-like 3	2.03 × 10^−3^	2.63
*Pctp*	phosphatidylcholine transfer protein	7.83 × 10^−4^	2.14
*Pik3c2g*	phosphatidylinositol 3-kinase, C2 domain containing, γ polypeptide	1.53 × 10^−5^	1.66
*Rbp1*	retinol binding protein 1, cellular	9.88 × 10^−5^	2.14
*Rdh11*	retinol dehydrogenase 11	1.70 × 10^−2^	1.5
*Rdh16*	retinol dehydrogenase 16	6.55 × 10^−5^	1.97
*Rdh9*	retinol dehydrogenase 9	8.30 × 10^−4^	2.24
*Sigmar1*	sigma non-opioid intracellular receptor 1	1.40 × 10^−4^	1.51
*Hmgcs1*	3-hydroxy-3-methylglutaryl-Coenzyme A synthase 1	1.42 × 10^−2^	1.65
*Mogat1*	monoacylglycerol *O*-acyltransferase 1	2.20 × 10^−4^	2.78
*Vldlr*	very low density lipoprotein receptor	5.65 × 10^−5^	2.45
**Inflammatory Response**			
*Cxcl1*	chemokine (C-X-C motif) ligand 1	1.64 × 10^−2^	0.65
*Mbl1*	mannose-binding lectin (protein A) 1	1.72 × 10^−4^	0.64
*Serpina3n*	serine (or cysteine) peptidase inhibitor, clade A, member 3N	4.95 × 10^−4^	0.58
*Saa1*	serum amyloid A 1	1.27 × 10^−5^	0.17
*Saa2*	serum amyloid A 2	4.27 × 10^−5^	0.17
*Saa4*	serum amyloid A 4	5.13 × 10^−5^	0.54
*C4a*	similar to Complement C4 precursor	5.89 × 10^−4^	0.64
*Stat3*	signal transducer and activator of transcription 3	2.60 × 10^−4^	0.65

The upregulated differentially expressed genes related to PPAR signaling pathway, including 10 repeating differentially expressed genes (Acaa1b, Acsl5, Cpt1b, Ehhadh, Fabp4, Lpl, Cyp4a31, Cyp4a10, Cyp4a32, Scd1) in Lipid metabolic process, and downregulated differentially expressed genes related to Inflammatory response are listed.

**Figure 4:**
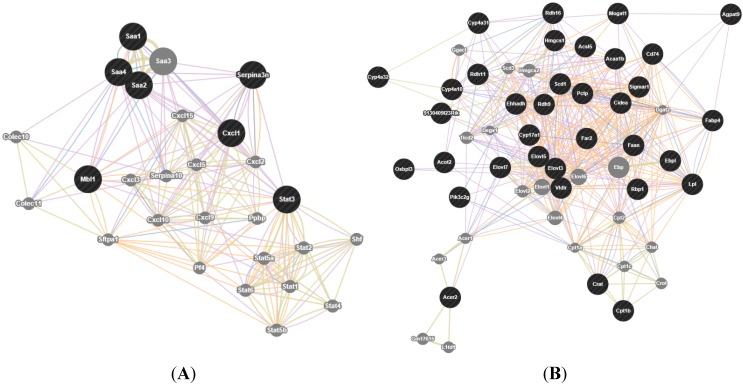
Gene network produced using GeneMania. (**A**) Gene network of inflammatory response, the network consists of 27 genes (circles) connected by 207 interactions (edges); (**B**) gene network of lipid metabolic process, the network consists of 55 genes (circles) connected by 442 interactions (edges). The interactions found between these genes are: co-expression (purple lines), co-localization (blue lines), shared protein domains (gray-yellow lines) and predicted (yellow lines). Genes that are within a black filled circle indicate differentially expressed genes, while those within a gray filled circle indicate their interactions.

To easily identify which genes were closely associated with metabolic disorders such as dysfunction of lipid metabolism and steatosis, gene network analysis was performed using GeneMania (Figure 4). As expected from a trait with highly polygenic architecture of lipid metabolic process and inflammatory response, there were a large number of genes involved in the network, with several connections. The number of interactions for Scd1, Elovl3, Fasn, Elovl5 and Fabp4 in the network was 41, 41, 35, 26, and 20 connections, respectively, and their potential role in determining the effect of pioglitazone is discussed below.

## 3. Discussion

In the present study, we demonstrated that administration of the insulin sensitizer, pioglitazone, could improve systemic insulin resistance, but exacerbated the development of fatty liver in the DIO mice which exhibited typical features of type 2 diabetes, such as hyperglycemia, hyperinsulinemia, insulin resistance, dyslipidemia and hepatic steatosis. We further performed global gene expression profiling of liver between pioglitazone-treated DIO mice and control DIO mice. These results indicated that a larger proportion of lipid metabolism and PPAR signaling pathway-related genes were upregulated. In contrast, the alterations in the downregulated differentially expressed genes were allocated to inflammatory response.

Our analysis revealed that genes encoding fatty acids uptake (Fabp4, Cd36) and *de novo* lipogenesis (Scd1, Fasn, Elovl3,5,7) were upregulated in the liver of pioglitazone-treated mice (Table 3). The GeneMANIA computer program also identified a tight network between those genes. The network included 55 genes with 442 interactions among them. (Figure 4B). Expression changes in these genes were consistent with the results that pioglitazone treatment exacerbated hepatic lipid accumulation (Figure 2). The balance between TG hydrolysis/secretion and TG uptake/synthesis is important to maintain lipid homeostasis in the liver. Clearly, it would be detrimental to lipid metabolism if any of these pathways were perturbed. In the case of NAFLD, hepatic steatosis can be stimulated via increased *de novo* lipogenesis and FFA uptake, or the decreased fatty acid beta oxidation and TG hydrolysis. PPARγ is most highly expressed brown adipose tissue (BAT) and white adipose tissue (WAT), where it serves as a key regulator of adipogenesis and a potent modulator of whole-body insulin sensitivity and lipid metabolism [22]. In fact, in the liver of DIO mice, pioglitazone treatment significantly increased mRNA levels of CD36 and Fabp4, which were well recognized target genes of PPARγ (Table 3). These genes are involved in fatty acid transportation and fat droplet deposition. Indeed it has been observed that the hepatic expression of CD36 and Fabp4 was positively correlated with hepatic TG contents in NAFLD patients, which give emphasis to the potential importance of these transporters for this disease [23,24]. These changes were consistent with the observation that hepatic steatosis exhibit increased liver PPARγ expression in mouse models [9,10,11,12]. Thus, our results suggest that pioglitazone-mediated PPARγ hyperactivity may lead to adipogenic hepatic steatosis and hepatic adiposis in DIO mice.

Furthermore, differentially expressed genes that were upregulated in progressors were also enriched for lipid biosynthetic processes (Table 1). Among these genes, fatty acid synthase (Fasn) and stearoyl-Coenzyme A desaturase 1 (Scd1) were of particular interest (Table 3 and Figure 4B). Fasn is a rate-limiting enzyme in the fatty acid biosynthesis and the last step in this pathway, while Scd1 is a microsomal enzyme that catalyzes the formation of monounsaturated long-chain fatty acids from saturated fatty acyl-CoAs, and is predominantly expressed in the liver [25,26]. In line with increased *de novo* lipogenesis in NAFLD, several studies have found that Scd1 and Fasn mRNA expression were significantly higher in NAFLD patients and high-fat mice [27,28]. Although Fasn and Scd-1 were not direct target genes of PPARγ, some studies have shown that overexpression of hepatocyte PPARγ and treatment with PPARγ agonists could promote expression of these genes [11,29], whereas ablation of PPARγ in the liver decreased their expression and eliminated the response to PPARγ agonists in obese mice [12,13]. The observation that pioglitazone enhance lipogenic gene expression in DIO mice in our study is consistent with these findings.

Collectively, our results indicate that pioglitazone simultaneously promotes hepatic uptake and *de novo* synthesis of FFA. All of these factors contribute to pioglitazone induced TG accumulation and the increase in microvesicular lipid droplets in the liver of DIO mice. However, it is reported that treatment with PPARγ agonists, including pioglitazone, improved hepatic steatosis in patients with NAFLD [5,6,7], as well as in other animal models of NAFLD [16,17,18,19]. Thus, an important question concerns the similarity between mouse and human hepatic steatosis need to be addressed. We are unaware of any published studies quantitating PPARγ levels in steatotic liver from humans. The levels of PPARγ in liver may be a key factor that determines whether a PPARγ agonist could induce PPARγ hyperactivity. Besides, the difference in the drug dosages used in animal experiment and clinical studies, as well as hepatic distribution of the drug, may also explain these discrepancies.

The downregulated genes in progressors were enriched for inflammatory and defense response genes (Table 1). Among these inflammatory genes, Saa(1,2,4), encoding a major acute-phase serum amyloid A protein [30], was strongly downregulated (Table 3). Serum amyloid A proteins is a proposed mediator of inflammation and metabolism, and its increased serum levels have been associated with obesity, chronic hyperglycemia, insulin resistance and cardiovascular disease [31,32,33,34]. Thus, SAA might be one of the potential factors linking chronic inflammation and the development of a metabolic syndrome. Our data indicate that SAA levels in patients dropped when insulin sensitivity was restored by treatment with a PPARγ agonist [35,36]. Adipose tissue shows the highest PPARγ expression and is the tissue with the most notable gene expression changes in response to treatment with PPARγ agonists [37]. Therefore, it is widely agreed that the insulin-sensitizing effects, as well as certain negative side effects of TZDs are the consequences of adipose-specific PPARγ activation. Conversely, the facts that PPARγ is expressed, though at lower levels, in a variety of non-adipose tissues and that TZDs improved insulin sensitivity in lipodystrophic (fatless) mice suggests that other tissues might also be direct targets and contribute to the insulin-sensitizing effects of TZDs, as well as to the side effects [12,38,39]. Moreover, pioglitazone exhibited an anti-inflammatory property in liver of DIO mice, which appeared to account for the insulin-sensitizing effect as the inflammatory status was a critical determinant of insulin sensitivity in hepatocytes [40], as well as other cells in liver such as macrophages and Kupffer cells [41,42].

## 4. Experimental Section

### 4.1. Animals and Treatment

Six-week-old male C57BL/6J mice (Institute of Laboratory Animal Science, CAMS and PUMC, Beijing, China) were housed in controlled temperature (22–25 °C), under 12 h-light/dark cycle and given food and water *ad libitum*. All animal experiments were conducted in accordance with The Standards for Laboratory Animals (GB14925-2001) and The Guideline on the Humane Treatment of Laboratory Animals (MOST 2006a) established by the People’s Republic of China. The two guidelines were observed in addition to the regulations of Institutional Animal Care and Use Committee (IACUC) and all animal protocols were approved by IACUC.

Mice were fed with a high-fat diet (60% of calories from fat, Research Diets, Inc., Beijing, China) for 16 weeks and were randomly divided into two groups (*n* = 12/group): the control group (0.5% CMC-Na) and pioglitazone group (25 mg/kg/day). Pioglitazone was given by oral gavage once a day for 38 days. Additionally, twelve age- and gender-matched C57BL/6J mice were given with standard chow (13% of calories from fat, Research Diets, Inc., Beijing, China) as normal group (Nor). Insulin tolerance test (ITT) was performed on day 30. At the end of the experiment, the mice (8 from each group) were decapitated and the livers were immediately excised and weighed, then stored at −80 °C for further analysis.

### 4.2. Biological Analysis and Insulin Tolerance Test

Blood samples were obtained from the tail-tip of mice fasted for 4 h. Plasma glucose levels were measured by the glucose oxidase methods, and insulin was measured by ELISA (AlPCO Inc., Salem, NH, USA). For the ITT, all animals were subcutaneously injected insulin at dose of 0.4 IU/kg. At 40 and 90 min after injection, blood was acquired for determination of plasma glucose levels. For liver lipids assay, a 50 mg aliquot of liver was homogenized and the lipids extracted. The levels of TG and FFA in liver tissues were determined using commercial kits (BioSino, Inc., Beijing, China, and Sekisui Medical, Tokyo, Japan) according to manufacturer’s instructions.

### 4.3. Hyperinsulinemic-Euglycemic Clamp Study

The clamp was performed at the end of treatment as described previously [43]. Animals were fasted for 12 h, anesthetized with sodium pentobarbital (50 mg/kg body weight, intraperitoneal injection) and placed on a heating pad at 37 °C. The right jugular vein was catheterized (Micro-renathane, 0.025 × 0.012 in.) for the infusion of glucose and insulin. Insulin was infused by a programmable syringe pump (Cole Parmer, Vernon Hills, IL, USA), and glucose was infused by a low-flow, high-accuracy pump (IPC, Ismatec, Switzerland). After the infusion, animals were rested for 30 min to decrease irritable responses. Then, a primed continuous infusion of insulin was given at a concentration of 20.0 milliunits/kg/min. Plasma glucose concentration was monitored instantaneously with the Accu-Chek active blood glucose monitoring meter and indicator papers (Roche Diagnostics, Mannheim, Germany) in order to maintain the basal level 6.0 ± 0.5 mmol/L by the perfusion of 10% glucose at variable rates. When the blood glucose had maintained a steady state for at least 20 min, the glucose infusion rate (GIR) was measured five times and a mean value was calculated.

### 4.4. Liver Histopathology

The livers were fixed in 4% paraformaldehyde, paraffin embedded and sectioned at a thickness of 2 μm. Tissue sections were stained with hematoxylin and eosin (H&E), and examined under a light microscope (Olympus CX41RF, Olympus, Tokyo, Japan) using standard protocols. 

### 4.5. Preparation of RNA and Microarray Hybridization

Total RNA was extracted from frozen liver tissue using TRIZOL Reagent (Life technologies, Carlsbad, CA, USA) following the manufacturer’s instructions. RNA quantity and quality were assessed by Nanodrop (Nanodrop, Wilmington, DE, USA). The 260/280 ratios of all samples were between 2.00 and 2.04. The integrity and quality of the RNA was assessed using the Bioanalyzer (Agilent Technologies, Santa Clara, CA, USA). All RNA integrity number (RIN) values were ≥7.0. Isolated RNA was further purified using an RNeasy Mini Kit (Qiagen, Hilden, Germany). Purified total RNA was amplified by *in vitro* transcription and converted to sense-strand cDNA using a WT Expression kit (Ambion/Applied Biosystems, Foster City, CA, USA). cDNA was fragmented and labeled using a GeneChip WT Terminal Labeling kit (Affymetrix, Santa Clara, CA, USA). Fragmented cDNA samples were then hybridized to GeneChip Mouse Gene 1.0 ST Arrays (Affymetrix, Santa Clara, CA, USA). Procedures were carried out as described by the manufacturers. Images were processed and GeneChip Command Console Software (Affymetrix) were used to generate cell intensity files (CEL files). CEL files were imported into Expression Console and normalized using robust multiarray average (RMA).

### 4.6. GeneChip Microarray Analysis

Raw data from gene chips were summarized using RMA, which involves quantile normalization. Genes showing a statistically significance (*p* < 0.05) and a log2-transformed fold change of at least ±1.5 were identified as differentially expressed. Microarray data were validated using qRT-PCR for 8 selected DEGs, which demonstrated high correlation between microarray and qRT-PCR expression levels.

The Database for Annotation, Visualization and Integrated Discovery (DAVID) v6.7 [22] was used to determine pathways and processes of major biological significance based on Gene Ontology (GO) categories over-represented among DEGs. Kyoto Encyclopedia of Genes and Genomes (KEGG) pathway tool was used to cluster of the genes involved in common pathways and processes for both pathway specific and molecular overview purposes. KEGG pathway tools were utilized through DAVID online tools. Differentially expressed genes related to lipid metabolic process and inflammatory response were used for performing gene network analysis using the online resource GeneMania.

### 4.7. Quantitative Real-Time PCR

The expression levels of eight DEGs (highly upregulated or downregulated in the DIO samples) were measured by quantitative Real-Time PCR (qRT-PCR) in six biological replicates in both Pioglitazone treatment group and Con group samples for technical validation of microarray data. Results were expressed as fold expression relative to expression in the Con group using the delta-delta Ct (ΔΔ*C*t) method. The level of β-actin RNA was used as an internal standard.

### 4.8. Statistical Analysis

Data are presented as means ± S.E.M. and were compared by one-way ANOVA with *post hoc* tests to vehicle treated DIO mice group. Outcomes of *p* < 0.05 were considered to be statistically significant.

## 5. Conclusions

In conclusion, according to our gene expression analysis of the liver from the pioglitazone-treated mice, genes regulate lipid metabolic process, and PPAR signaling pathway were upregulated, while genes related to inflammation response were downregulated. These changes were consistent with the effect of chronic treatment with pioglitazone, such as ameliorating systematic insulin resistance and exacerbating hepatic steatosis in DIO mice. Importantly, it is possible that the deterioration of hepatic steatosis with pioglitazone treatment in obese DIO mice was a species-specific manifestation, similar to the hepatomegaly caused by PPARα agonists in rodents [44]. Therefore, T2DM patients with NAFLD should be aware of the side effects of TZDs administration. Our findings suggested that large scaled, well-controlled and long-term clinical trials are necessary to assess the long-term clinical benefits of pioglitazone for NAFLD patients.
